# Supplementary material for: The interactome of intact mitochondria by cross-linking mass spectrometry provides evidence for coexisting respiratory supercomplexes
Source: Mol Cell Proteomics. 2017 Dec 8;17(2):216–32. doi: 10.1074/mcp.RA117.000470 (PMC5795388; doi:10.1074/mcp.RA117.000470)
Supplement: Supplemental Data [file supp_RA117.000470_133922_0_supp_23978_fzffwf.zip › spectra_annotation/mito_spectra_annotation/97-1-8-1-2-1.pdf]

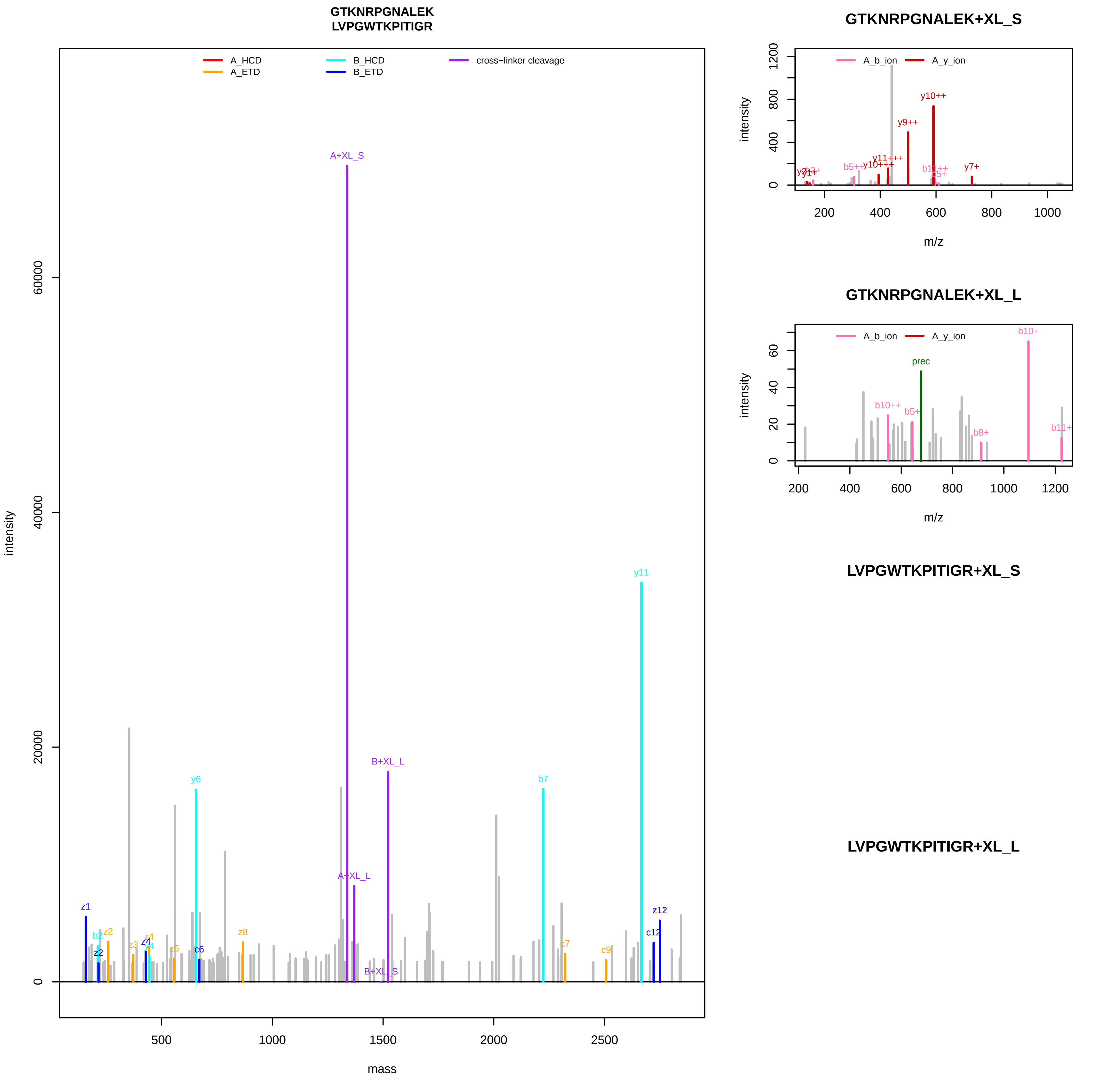
GTKNRPGNALEK
LVPGWTKPITIGR
A_HCD
A_ETD
B_HCD
B_ETD
cross−linker cleavage
A+XL_S
A+XL_L
B+XL_L
B+XL_S
y11
y6
b7
z1
b2
z2
z3
z4
y4
z5
c6
z8
c7
c9
c12
z12
intensity
mass
0
20000
40000
60000
500
1000
1500
2000
2500
GTKNRPGNALEK+XL_S
A_b_ion
A_y_ion
y10++
y9++
y11+++
y10+++
b5++
y7+
y1+
m/z
intensity
0
400
800
1200
200
400
600
800
1000
GTKNRPGNALEK+XL_L
A_b_ion
A_y_ion
b10+
prec
b10++
b5+
b8+
b11+
m/z
intensity
0
20
40
60
200
400
600
800
1000
1200
LVPGWTKPITIGR+XL_S
LVPGWTKPITIGR+XL_L
